# Evolving Therapeutic Strategies in ANCA-Associated Vasculitis: Current Standards and Emerging Targets for GPA and MPA

**DOI:** 10.1007/s12016-026-09147-5

**Published:** 2026-03-23

**Authors:** Roberto Dal Pozzolo, Luca Iorio, Federica Davanzo, Elisabetta Zanatta, Luca Iaccarino, Roberta Ramonda, Roberto Gerli, Andrea Doria, Giacomo Cafaro, Roberto Padoan

**Affiliations:** 1 Rheumatology Unit, Department of Medicine, University of Perugia, Perugia, Italy; 2 Rheumatology Unit, Department of Medicine DIMED, University of Padua, Padua, Italy

**Keywords:** ANCA-associated vasculitis, Granulomatosis with polyangiitis, Microscopic polyangiitis, Rituximab, Complement inhibition, C5a receptor antagonist, Glucocorticoid-sparing therapy, Refractory vasculitis

## Abstract

Granulomatosis with polyangiitis (GPA) and microscopic polyangiitis (MPA) are severe autoimmune disorders characterized by necrotizing small-vessel inflammation, and are classified among anti-neutrophil cytoplasmic antibody (ANCA)-associated vasculitides (AAV). Standard induction therapy combines glucocorticoids (GCs) with rituximab (RTX) or cyclophosphamide (CYC), with growing emphasis on GC minimization and selective use of avacopan in patients at high risk of GC toxicity. For maintenance therapy, fixed-interval RTX generally outperforms conventional oral agents and biomarker-guided re-dosing in unselected populations, yet treatment should be individualized. Persistent challenges include treatment-related toxicity, refractory manifestations, and defining safe discontinuation strategies. Expanding knowledge of AAV immunopathogenesis has driven the development of novel, mechanism-based therapies. These include agents targeting B cells and plasma cells (anti-CD38, anti-CD19, proteasome inhibition, CAR-T cells), complement components, and T-cell co-stimulation or cytokine networks (abatacept, IL-6 and JAK-inhibitors). Collectively, these advances are shifting AAV care from broad immunosuppression toward precision immunotherapy aimed at durable remission with reduced GC exposure and minimized long-term toxicity.

## Introduction

Anti-neutrophil cytoplasmic antibody (ANCA)-associated vasculitides (AAV) are rare, chronic autoimmune diseases characterized by pauci-immune necrotizing inflammation predominantly affecting small- to medium-sized blood vessels, leading to multi-organ involvement [1]. AAV comprises three major clinicopathologic entities: granulomatosis with polyangiitis (GPA), microscopic polyangiitis (MPA), and eosinophilic granulomatosis with polyangiitis (EGPA) [2]. GPA is most frequently associated with antibodies targeting leukocyte proteinase-3 (PR3), which typically display a cytoplasmic ANCA (c-ANCA) pattern at indirect immunofluorescence [3]. In contrast, antibodies directed against myeloperoxidase (MPO), usually associated with a perinuclear ANCA (p-ANCA) pattern, predominate in MPA and in a subset of EGPA patients. Although categorized as a form of AAV, EGPA differs substantially in terms of genetic background, pathogenesis, clinical manifestations, and therapeutic approach and is generally considered a separate disease entity [4]. Accordingly, this review will focus on GPA and MPA, which share greater clinical and pathogenetic similarities and are often grouped together in clinical studies [5]. Despite this overlap, GPA is typically characterized by granulomatous inflammation and frequent ear,nose, and throat (ENT) involvement, whereas MPA more commonly presents with severe renal disease and necrotizing vasculitis without granulomas [3]. Over recent decades, the therapeutic landscape of AAV has evolved substantially. The introduction of glucocorticoids (GCs), cyclophosphamide (CYC) and more recently rituximab (RTX), has transformed AAVs from potentially fatal diseases into chronic, but manageable conditions. Nevertheless, relapses remain common [6] and long-term management requires careful balancing of disease control against treatment-related toxicity. RTX has emerged as a cornerstone of maintenance therapy due to its efficacy in sustaining remission and favourable safety profile compared with CYC [7], although optimal dosing strategies, duration of therapy, and monitoring approaches remain areas of ongoing investigation [8–11]. This review provides an updated overview of established and emerging immunotherapeutic strategies for AAV, highlighting their mechanisms of action, clinical efficacy, limitations, and future perspectives.

## Current Treatment Strategies for AAV: Insights from EULAR, ACR, and KDIGO Recommendations

The international recommendations from the European Alliance of Associations for Rheumatology (EULAR), the American College of Rheumatology/Vasculitis Foundation (ACR/VF), and the Kidney Disease: Improving Global Outcomes (KDIGO) clinical practice guidelines provide a broadly shared approach for managing AAV, recommending an induction phase aimed at rapidly controlling inflammation and preventing irreversible organ damage, followed by a longer maintenance phase to sustain remission [12–14]. For life- or organ-threatening disease, remission induction typically involves GCs combined with either RTX or CYC. Oral GCs should be initiated immediately upon diagnosis, with different regimens evolving toward steroid-sparing strategies. IV methylprednisolone (MPN) pulses may be used in selected cases, although there is no clear consensus regarding their routine use [15]. Regarding immunosuppressive therapy, both ACR/VF and EULAR prefer RTX over CYC due to its comparable efficacy, lower toxicity, and better capacity to sustain remission in relapsing disease [7, 12, 13]. In contrast, KDIGO recommends CYC as the preferred agent if patients present with markedly reduced or rapidly declining estimated glomerular filtration rate (eGFR) and supports combining RTX and CYC in selected cases [14]. This strategy has shown encouraging results, with evidence suggesting a reduction in end-stage kidney disease (ESKD) and mortality, enhanced remission rates, and a potential to minimize toxicity associated with both GCs and CYC. However, evidence on its long-term safety is still limited [16–19]. A randomized trial (ENDURRANCE) is currently underway to further investigate these aspects [20]. The role of plasma exchange (PLEX) remains a point of debate. While early studies, including one randomized trial (MEPEX), suggested potential benefits in patients with severe renal involvement (specifically those with serum creatinine > 5.8 mg/dL [> 500 µmol/L]) [21], a larger randomized controlled trial (PEXIVAS) failed to demonstrate a significant reduction in mortality or progression to ESKD at 12 months [22, 23]. By contrast, a recent systematic review and meta-analysis of randomized controlled trials (nine trials overall; seven reporting ESKD) found that PLEX probably reduced the risk of ESKD at 12 months (RR 0.62, 95% CI, 0.39–0.98; moderate certainty of evidence) [22]. Reflecting this mixed evidence, EULAR recommends PLEX in selected cases with severe renal disease, with serum creatinine > 3.4 mg/dL (> 300 µmol/L) [12]. ACR/VF advises against its routine use, reserving it for critically ill patients unresponsive to initial treatment [13]. In contrast, KDIGO adopts a more permissive stance, suggesting consideration of PLEX in patients with serum creatinine > 3.4 mg/dL (> 300 µmol/L), those with rapidly progressive renal failure or requiring dialysis, and in cases of diffuse alveolar haemorrhage (DAH) with hypoxemia [14]. The pivotal role of complement in the pathogenesis of AAV has driven the development of targeted therapies [23]. All three complement pathways - classical, lectin, and alternative - culminate in C5 convertase, which cleaves C5 to release the anaphylatoxin C5a, a key effector molecule with strong pro-inflammatory activity. C5a acts as a neutrophil primer, enhancing ANCA responsiveness and perpetuating the inflammatory cascade [24]. By antagonizing the C5a receptor, avacopan has emerged as a novel therapeutic option, offering an alternative to high-dose GCs in induction regimens, and is now included in the latest EULAR and KDIGO guidelines. In a randomized, controlled phase 3 trial (ADVOCATE), avacopan was non-inferior to a GC taper for remission at week 26 and superior for sustained remission at week 52, while substantially reducing cumulative GC exposure and GC-related toxicity, including lower infection rates [25, 26]. Beyond its GC-sparing effect, avacopan demonstrated a clinically meaningful renal benefit, with a consistent renal recovery signal characterized by greater improvement in eGFR and a faster reduction in albuminuria compared with in the control group [26]. Subgroup analyses suggest that patients with severe renal involvement, particularly those with markedly reduced baseline eGFR, derive the greatest benefit, supporting the use of avacopan when renal recovery and avoidance of GC toxicity are concurrent therapeutic priorities [26]. Finally, supplementary analyses supported the efficacy of avacopan for achieving remission in patients with relapsing disease [26]. Emerging real-world evidence extends the potential utility of avacopan beyond the trial population, supporting its use in patients with rapidly progressive glomerulonephritis, including those requiring dialysis, and in AAV-related pulmonary haemorrhage, where encouraging outcomes have been reported [27, 28]. Despite these promising results, important evidence gaps remain, including limited long-term follow-up beyond 12 months, residual uncertainty regarding GC-sparing regimens, and the need for clearer identification of patient subgroups most likely to derive clinical benefit. For non-organ-threatening disease, RTX continues to be the preferred option, with methotrexate (MTX) or mycophenolate mofetil (MMF) as alternatives in selected cases. Nevertheless, these agents have been associated with higher relapse rates [29, 30]. Once remission is achieved, the therapeutic focus shifts to maintenance, essential to prevent relapses. Regardless of the induction regimen used, RTX has become the standard of care for sustaining remission, as alternatives such as azathioprine (AZA), MMF, or MTX (in patients with eGFR > 60 mL/min/1.73 m²) are associated with a higher risk of relapse [31]. The duration of maintenance therapy varies across guidelines, ranging from a minimum of 18 months to up to 4 years, and should be tailored to the individual patient’s relapse risk. The key clinical trials in AAV treatment are briefly summarized in Table 1.

**Table 1:** Summary of key data from the landmark trials in AAV

Trial	Intervention	Population		Treatment arm	Main outcome	Key results	Relapses
Induction of Remission							
NORAM (Nonrenal Wegener’s Granulomatosis Treated Alternatively with Methotrexate) 2005 [32]	MTX vs. CYC		*N* = 100	• Arm 1: MTX 20–25 mg/week plus PDN (*n* = 51) • Arm 2: Daily CYC 2 mg/kg/day plus PDN (*n* = 49)	Non inferiority for induction remission	• Arm 1: 44/49 (89.9%) patients achieved remission at 6 months • Arm 2: 43/46 (93.5%) patients achieved remission at 6 months The null-hypothesis of inferiority by more than 15% was rejected, *P* = 0.041	• Arm 1: 32/46 (69.5%) of patients • Arm 2: 20/43 (46.5%) of patients
MEPEX (Methylprednisolone Plasma Exchange) 2007 [21]	PLEX vs. IV MPN (on top CYC)		*N* = 137	• Arm 1: PLEX plus oral CYC plus PDN (*n* = 70) • Arm 2: IV MPN 1 g/day for 3 days plus oral CYC plus PDN (*n* = 67)	Renal recovery in severe renal vasculitis	• Arm 1: 48/70 (69%) of patients • Arm 2: 33/67 (49%) of patients (95% CI, 18 to 35%); *P* = 0.02	-
CYCLOPS (Cyclophosphamide Daily Oral versus Pulsed) 2009 [33]	Oral CYC vs. IV CYC		*N* = 149	• Arm 1: IV CYC 15 mg/kg every 2–3 weeks plus PDN (*n* = 76) • Arm 2: CYC 2 mg/kg/day plus PDN (*n* = 73)	Time to remission	• Arm 1: median time to remission 3 [0.5–8.5] months • Arm 2: median time to remission 3 [1–7.5.5] months The groups did not differ in time of remission HR 1.098 (95%, CI, 0.78 to 1.55), *P* = 0.59	• Arm 1: 13 (7 major and 6 minor) relapses • Arm 2: 6 (3 major and 3 minor) relapses
RAVE (Rituximab in ANCA-associated Vasculitis) 2010 [34]	RTX vs. oral CYC		*N* = 197	• Arm 1: RTX 375 mg/m2/week for 4 weeks (*n* = 99) • Arm 2: CYC 2 mg/kg/day and AZA 2 mg/kg/day as maintenance (*n* = 98)	Remission (BVAS = 0) without PDN at 6 months	• Arm 1: 63/99 (64%) of patients • Arm 2: 52/98 (53%) of patients The treatment difference of 11% points between groups met the criterion of non-inferiority, *P* < 0.001	• Arm 1: 6 severe relapses (13 limited relapses) • Arm 2: 10 severe relapses (15 limited relapses) The rates of severe relapse were 0.11 and 0.018 per patient-month, respectively (*P* = 0.30)
RITUXVAS (Rituximab in Vasculitis) 2010 [35]	CYC + RTX vs. CYC + AZA		*N* = 44	• Arm 1: RTX 375 mg/m2/week for 4 weeks plus 2 IV CYC 15 mg/kg (*n* = 33) • Arm 2: IV CYC 15 mg/kg for 3 to 6 months and AZA 2 mg/kg/day as maintenance (*n* = 11)	Sustained remission rates at 12 months and SAE	• Arm 1: 25/33 (76%) of patients (6 patients died, 93% of the patients in the rituximab group had sustained remission) • Arm 2: 9/11 (82%) of patients (1 patient died, 90% of the patients in the control group had sustained remission) The absolute difference in sustained remission with Arm 1 as compared with Arm 2 was − 6% points (95% CI, − 33 to 21), *P* = 0.68 (among survivors *P* = 0.80)	• Arm 1: 4/27 (15%) of patients • Arm 2: 1/10 (10%) of patient *P* = 0.70
MYCYC (MYcophenolate vs. CYClophosphamide) 2019 [30]	MMF vs. IV CYC		*N* = 140	• Arm 1: MMF 2 g/day plus GC (*n* = 70) • Arm 2: IV CYC 15 mg/kg every 2–3 weeks plus GC and AZA 2 mg/kg/day as maintenance (*n* = 70)	Remission by 6 months requiring compliance with the GC tapering regimen	• Arm 1: 47/70 (67%) of patients • Arm 2: 43/70 (61%) of patients RD 5.7% (90% CI, − 7.5% to 19%) Non-inferiority margin of − 12%, the lower bound of the 90% CI of - 7.5%	• Arm 1: 23/63 (4 major and 19 minor) relapses • Arm 2: 13/64 (3 major and 10 minor) relapses IRR 1.97, 95% CI, 0.96 to 4.23, *P* = 0.049
PEXIVAS (Plasma Exchange and glucocorticoid In severe VASculitis) 2020 [36]	PLEX vs. no PLEX and reduced GC vs. standard GC		*N* = 752	• Arm 1: seven PLEX within 14 days plus standard therapy(CYC or RTX) (*n* = 352) • Arm 2: no PLEX plus standard therapy (CYC or RTX) (*n* = 352) And then randomized to • Arm R: reduced dose of oral GC regimen (*n* = 353) • Arm S: standard dose of oral GC regimen (*n* = 351)	Primary composite outcome of death from any cause or ESKD	PLEX: • Arm 1: 100/352 (28.4%) of patients • Arm 2: 109/352 (31.0%) of patients HR with PLEX 0.86 (95% CI, 0.65 to 1.13), *P* = 0.27 GC: • Arm R: 92/330 (27.9%) of patients • Arm S: 83/325 (25.5%) of patients Arm R non-inferior to Arm S, absolute RD, 2.3% points (90% CI, − 3.4 to 8.0; 95% CI, − 4.5 to 9.0)	204 relapses in 147 (22.7%) patients (IR 10.3 relapses per 100 patient-years; 95% CI, 8.4–12.1 per 100 patient-years) No evidence of an interaction between PLEX and the GC regimen (*P* = 0.34)[37]
LoVAS (Low dose GC in VASculitis) 2021 [38]	Low GC vs. High GC		*N* = 140	• Arm 1: Low dose GC (PDL 0.5 mg/kg/day stopped at 5 months) regimen plus RTX (*n* = 70) • Arm 2: High dose GC (PDL 1 mg/kg/day tapered to 5–10 mg/day) regimen plus RTX (*n* = 70)	Remission rate at 6 months	• Arm 1: 49/69 (71.0%) of patients • Arm 2: 45/65 (69.2%) of patients The difference was 1.8% points (1-sided 97.5% CI, − 13.7 to ∞) between groups, met the criterion for noninferiority, *P* = 0.003	• Arm 1: 3 (1 major and 2 minor) relapses • Arm 2: 0 relapse *P* = 0.24
ADVOCATE (Avacopan in Patients With AAV) 2021 [26]	Avacopan vs. oral PDN		*N* = 331	• Arm 1: Avacopan 30 mg twice dailyplus standard therapy (CYC or RTX) ((*n* = 166) • Arm 2: fixed tapered PDN plus standard therapy (CYC or RTX) (*n* = 165)	Clinical remission at week 26 and sustained remission at 52 week and no GC	Clinical remission: • Arm 1: 120/166 (72.3%) of patients • Arm 2: 115/164 (70.1%) patients Estimated difference, 3.4% points (95% CI, − 6.0 to 12.8), *P* < 0.001 for non-inferiority; *P* = 0.24 for superiority Sustained remission: • Arm 1: 109/166 (65.7%) of patients • Arm 2: 90/164 (54.9%) patients Estimated difference, 12.5% points (95% CI, 2.6 to 22.3), *P* < 0.001 for noninferiority; *P* = 0.007 for superiority	• Arm 1: 9/120 (7.5%) of patients at week 26 • Arm 2: 14/115 (12.2%) patients HR for relapse after remission (Arm 1 vs. Arm 2) was 0.46, 95% CI, 0.25 to 0.84
Maintenance of Remission							
CYCAZAREM (CYClophosphamide versus AZAthioprine for early REMission phase in AAV) 2003 [39]	Oral CYC vs. AZA	*N* = 144		• Arm 1: AZA 2 mg/kg/day plus PDL 10 mg (*N* = 71) • Arm 2: CYC 1.5 mg/kg/day plus PDL 10 mg (*N* = 73) At 12 months, both arms received AZA 2 mg/kg/day plus PDL 10 mg	Relapse, either major or minor	• Arm 1: 11/71 (15.5%), (5 major) of patients had a relapse • Arm 2: 10/73 (13.7%) (5 major) of patients had a relapse Difference 1.8% (95% CI, −9.9-13.0), *P* = 0.65	-
WEGENT (the Wegener’s Granulomatosis–Entretien trial) 2008 [40]	AZA vs. MTX	*N* = 126		• Arm 1: AZA 2 mg/kg/day (*N* = 63) • Arm 2: MTX 0.3 mg/kg/week (progressively increased every week by 2.5 mg, to 25 mg/week) (*N* = 63)	An AE requiring discontinuation of the study drug or causing death	• Arm 1: 7/63 (11%) of patients • Arm 2: 12/63 (19%) of patients *P* = 0.21, The HR for Arm 2 vs. Arm 1 was 1.65 (95% CI, 0.65 to 4.18), *P* = 0.29	• Arm 1: 23/63 (36%) of patients • Arm 2: 21/63 (33%) of patients *P* = 0.71
IMPROVE (International Mycophenolate mofetil Protocol to Reduce Outbreaks of Vasculitides) 2010 [41]	MMF vs. AZA	*N* = 156		• Arm 1: AZA 2 mg/kg/day (*N* = 80) • Arm 2: MMF 2 g/day (*N* = 76)	Relapse-free survival	Adjusted HR for relapses associated with Arm 2 of 1.80 (95% CI, 1.10–2.93), *P* = 0.02	• Arm 1: 30/80 (37.5%) of patients (10 with major and 20 with minor relapses) • Arm 2: 42/76 (55.2%) of patients (18 with major and 24 with minor relapses)
MAINRITSAN 1 (MAINtenance of Remission Using RITuximab in Systemic ANCA-associated vasculitis) 2014 [42]	RTX vs. AZA	*N* = 115		• Arm 1: RTX 500 mg every 6 months for 18 months (*N* = 57) • Arm 2: AZA 2 mg/kg/day for 22 months (*N* = 58)	The rate of major relapse at month 28	• Arm 1: 3/57 (5%) of patients • Arm 2: 17/58 (29%) of patients HR 6.61 (95% CI, 1.56 to 27.96), *P* = 0.002	• Arm 1: 6/57 (11%) of patients had a minor relapse • Arm 2: 9/58 (16%) of patients had a minor relapse *P* = 0.43
MAINRITSAN 2 (MAINtenance of Remission Using RITuximab in Systemic ANCA-associated vasculitis) 2018 [43]	Fixed RTX vs. B-cell/ANCA on demand RTX	*N* = 162		• Arm 1: RTX 500 mg every 6 months for 18 months (*N* = 81) • Arm 2: one fixed RTX 500 mg and then RTX 500 mg when B-cell or ANCA reappeared or rose markedly (*N* = 81)	Number of relapses at month 28	• Arm 1: 8/81 (9.9%) of patients • Arm 2: 14/81 (17.3%) of patients *P* = 0.22	-
MAINRITSAN 3 (MAINtenance of Remission Using RITuximab in Systemic ANCA-associatedvasculitis) 2020 [44]	Fixed RTX vs. placebo	*N* = 97		After completing a 18-month maintenance regimen • Arm 1: RTX 500 mg every 6 months for 18 months (*N* = 50) • Arm 2: placebo every 6 months for 18 months (*N* = 47)	Relapse-free survival at month 28	• Arm 1: 96% (95% CI, 91% to 100%) • Arm 2: 74% (CI, 63% to 88%) The absolute difference of 22% (CI, 9% to 36%), HR 7.5 (CI, 1.67 to 33.7), *P* = 0.008	-
RITAZAREM (RITuximab versus AZAthioprine for maintenance of REMission) 2023 [31]	RTX vs. AZA	*N* = 170		• Arm 1: RTX 1 g every 4 months for 5 doses (*N* = 85) • Arm 2: AZA 2 mg/kg/day for 24 months (*N* = 85)	Time from randomisation to disease relapse (major and minor)	Arm 1 was superior to Arm 2 for the prevention of major or minor disease relapse: HR 0.41 (95% CI, 0.27 to 0.61), *P* < 0.001	• Arm 1: 38/85 (45%) of patients had 52 relapses (11 major and 41 minor) • Arm 2: 60/85 (71%) of patients had 89 relapses (28 major and 61 minor)
MAINTANCAVAS (MAINTtenance of ANCA VASculitis remission) 2024 [45]	B-cell on demand RTX vs. ANCA on demand RTX	*N* = 115		After completing at least 24-months of fixed-schedule rituximab • Arm 1: RTX 1000 mg when B-cell reappeared (*N* = 58) • Arm 2: RTX 1000 mg x2 doses when ANCA reappeared or rose markedly (*N* = 57)	Clinical Relapse	• Arm 1: 4.1% (95% CI, 1.0 to 15.6) of patients • Arm 2: 20.5% (95% CI, 11.9 to 34.1) of patients At 3 years, log-rank *P* = 0.045	• Arm 1: 5/58 (8.6%) of patients • Arm 2: 14/57 (24.5%) of patients HR 0.37 (95% CI, 0.15 to 0.90).

*AAV* ANCA-Associated Vasculitis, *ANCA* Anti-Neutrophil Cytoplasmic Antibodies, *AE* Adverse Event, *AZA* Azathioprine, *BVAS* Birmingham Vasculitis Activity Score, *CI* Confidence Interval, *CYC* Cyclophosphamide, *ESKD* End Stage Kidney Disease, *GCs* glucocorticoids, *HR* Hazard Ratio, *IR* Incidence Rate, *IRR* Incidence Rate Ratio, *IV* Intravenous, *MMF* Mycophenolate Mofetil, *MPN* Methylprednisolone, *MTX* Methotrexate, *PDL* Prednisolone, *PDN* Prednisone, *PLEX* Plasma Exchange, *RD* Risk Difference, *SAE* Serious Adverse Events

## Persistent Challenges in AAV Treatment

Despite substantial therapeutic advances, key clinical controversies persist in the management of AAV, reflecting the complexity and evolving nature of treatment decision-making. As illustrated in Table 2, unresolved issues include the optimal strategy for RTX maintenance and discontinuation, as well as the appropriate role of avacopan and PLEX. In addition, major concerns persist regarding treatment-related toxicity, relapse risk, and refractory disease manifestations.

**Table 2:** From controversies to decisions in AAV

Question	What we know	Practical decision
Fixed vs. biomarker-guided RTX?	Fixed shows fewer relapses overall	Use **fixed q6 months** for most; reserve tailored for special cases
Avacopan for all?	Reduces GC exposure; benefits largest in GC-toxic risk	Use in patients where GC minimization is key and severe RPGN
PLEX in RPGN?	No mortality benefit; infection risk ↑	Consider in selected cases: SCr ≥ 3.4 mg/dL (> 300 µmol/L), RPGN/dialysis, and/or DAH with hypoxemia, after individualized risk–benefit discussion
When to stop RTX?	Some low-risk phenotypes safe to stop	Consider after **18–24 months** quiescence and close follow-up

*DAH* diffuse alveolar haemorrhage, *GC* glucocorticoid, *PLEX* plasma exchange, *RPGN* rapidly progressive glomerulonephritis, *RTX* rituximab, *SCr* serum creatinine

### Treatment-Related Toxicities

Clinical data from both trials and observational studies indicate that early and long-term mortality is more strongly associated with treatment-related complications than with disease activity itself [46–48]. 

High cumulative doses of GCs, used in both induction and maintenance therapy, are associated with significant complications in AAV patients, including diabetes, dyslipidaemia, weight gain, Cushingoid features, peptic ulcer, osteoporosis, cataracts, and psychiatric disorders [48–50]. Moreover, longer duration of GC exposure (more than 6 months) has been associated with increased cumulative damage in AAV [49, 51]. In recent years, increasing efforts have focused on GC minimization strategies, leading to the adoption of reduced-dose oral GC regimens that maintain efficacy while reducing treatment-related toxicity, despite heterogeneity in tapering schedules [38, 50, 52, 53]. Two randomized controlled trials (PEXIVAS and LoVAS) demonstrated that rapidly tapering GC regimens were non-inferior to standard regimens with regard to the primary outcomes of ESKD or death, with a reduction in severe infections of up to 30% in the reduced-dose GC arm [26, 38]. These findings align with those of a recent meta-analysis [54]. Combining avacopan with reduced GC dosing has also proven effective, as shown above [26]. Indeed, patients receiving avacopan with reduced GCs experience improved quality of life, better renal recovery, faster reduction in albuminuria, and predictably less GC-related toxicity [55]. 

CYC therapy is associated with numerous complications, including malignancies (e.g., urothelial cancer and non-melanoma skin cancer, among others), bone marrow failure, myelodysplasia, haemorrhagic cystitis, infections, nausea, vomiting, alopecia, hepatotoxicity, cardiotoxicity and permanent ovarian failure [56–59]. Over the past two decades, the European Vasculitis Study Group (EUVAS) has focused on minimising CYC exposure. CYC is a major contributor to malignancy risk in patients with AAV through its direct carcinogenic effects, with risk closely related to cumulative dose and a threshold of 36 g identified as clinically relevant [60]. EUVAS studies demonstrated that intravenous administration allows for reduced cumulative CYCexposurer while maintaining efficacy [33]. At 5-year follow-up, trial participants showed an increased risk limited to non-melanoma skin cancer, in contrast to with earlier reports [61, 62]. Lower cumulative CYC exposure and intravenous administration were associated with a reduced risk of leukopenia, while pre-hydration and mesna (sodium 2-mercaptoethanesulfonate) were effective in preventing bladder toxicity. Mesna is a detoxifying agent that inactivates acrolein, a toxic metabolite of CYC implicated in haemorrhagic cystitis and associated with an increased risk of bladder cancer [33, 63]. Nonetheless, a French study indicated that the risk of urothelial cancer persists even with low doses of CYC [64]. Long-term follow-up still showed high relapse rates and unfavourable outcomes [61, 62]. Higher cumulative CYC exposure, particularly when combined with older age at treatment initiation, is associated with an increased risk of primary ovarian failure and amenorrhoea. Studies across multiple autoimmune diseases report gonadal failure rates of 12–78%, with azoospermia approaching 60% [65]. In contrast, the risk of developing gonadal failure after a single course of CYC induction treatment for AAV is reported to be less than 5% [66–68]. Consequently, pre-treatment counselling is essential. Finally, CYC, along with MTX and MMF, demonstrates teratogenicity. Women receiving these agents must be adequately informed about potential reproductive risks and strongly advised to use effective contraception throughout treatment and for 3–6 months after treatment cessation. By contrast, AZA is regarded as having a favourable pregnancy safety profile, with no meaningful increase in major congenital malformations, as supported by EULAR recommendations [69–71]. 

RTX approval marked a significant advancement in the treatment of AAV, enabling a reduction in CYC-related toxicity [72, 73]. However, RTX has drawbacks, including treatment failures and adverse events. As with other medications, hypersensitivity reactions occur in approximately one-third of AAV patients, manifesting as urticaria, diffuse swelling, and anaphylaxis [74–76]. Notably, human anti-chimeric antibodies (HACA) have been linked to severe infusion reactions or RTX-induced serum sickness (RISS) following repeated administration [77]. Hypogammaglobulinemia occurs frequently in RTX-treated patients, affecting approximately 40–60% of cases, typically within the first six months of therapy, and is usually mild and transient [78–81]. However, lower IgG levels correlate directly with the development of serious infections. In a retrospective study involving a cohort of 239 RTX-treated AAV patients, 4.6% developed IgG levels below 4 g/L. Serious infections, though rare during maintenance therapy (0.85 per 10 patient-years; 95% CI, 0.66–1.1), were independently associated with IgG levels below 4 g/L [78, 82]. Late-onset neutropenia (LON), defined as neutrophil counts below 1.5 × 10⁹/L occurring at least four weeks after the last infusion, has been observed in RTX-treated AAV patients. LON is an idiosyncratic condition with no clearly identified predisposing factors. It affects 12–23% of patients and, while often self-limiting, sometimes leads to infectious complications [83, 84]. The biological effects of RTX (namely, B-cell depletion) persist long after drug elimination. Its mean half-life is 18–22 days, with near-complete elimination estimated within 3–4 months [85]. This prolonged effect raises two significant concerns: vaccination and pregnancy. EULAR guidelines recommend administering vaccinations before initiating RTX whenever possible, as several studies demonstrate suboptimal vaccination response during RTX treatment [86, 87]. Regarding pregnancy and reproductive safety, updated EULAR recommendations consider RTX compatible with use prior to conception and, when clinically necessary for severe or organ-threatening disease, during pregnancy following an individualized risk-benefit assessment [71, 88]. Placental transfer of IgG1 monoclonal antibodies is minimal in early gestation but increases later in pregnancy [88]. Therefore, second- or third-trimester exposure to RTX may result in transient neonatal B-cell depletion and/or cytopenias, with recovery typically occurring within the first months of life. When late-pregnancy exposure occurs, postnatal monitoring is advised and live vaccinations should be deferred until immune reconstitution. Overall, available observational data have not shown an increased risk of major congenital malformations, although neonatal cytopenias have been reported [71, 85, 89]. In AAV specifically, pregnancy-related safety data remain limited, underscoring the importance of shared decision-making and multidisciplinary management. As with other immunosuppressive therapies, infections represent the main safety concern following RTX therapy in AAV patients. Serious infections occur more frequently during remission induction, than during maintenance therapy [90, 91]. Bacterial, viral, and fungal infections have been reported, with lower respiratory tract infections and Pneumocystis jirovecii pneumonia being the most common [92–94]. Consequently, prophylaxis against Pneumocystis jirovecii pneumonia is recommended during RTX treatment [12]. 

Finally, as discussed above, the role of PLEX in AAV remains a matter of debate [95, 96]. Data summarized in a meta-analysis of four randomized controlled trials, including a total of 908 participants, showed that PLEX was associated with an increased risk of serious infections at 12 months (RR 1.27, 95% CI, 1.08–1.49; moderate certainty of evidence) [22]. This corresponds to an absolute increase in serious infections of 8.6% among patients with baseline serum creatinine levels between 300 and 500 µmol/L and 13.5% among those with creatinine levels > 500 µmol/L. No significant effect on health-related quality of life was observed. However, the same meta-analysis found no important effect of PLEX on all-cause mortality (RR 0.90, 95% CI, 0.64–1.27; moderate certainty of evidence) [22]. 

### Relapse Risk

Despite the high efficacy of currently recommended induction therapies, AAV remains a relapsing–remitting disease, with fluctuations in disease activity representing part of its natural course, and approximately 10–12% of patients developing refractory disease, often defined as failure to achieve remission after standard induction [94, 97, 98]. Failed remission and relapse rates observed across induction and maintenance trials are summarized in Table 1. Consequently, achieving durable long-term remission continues to represent a major unmet clinical need. In particular, the optimal strategy for maintaining remission and preventing relapses has yet to be clearly defined [74, 99]. As previously mentioned, the initial EUVAS trials successfully achieved their goal of minimizing CYC exposure while maintaining comparable remission rates. Nonetheless, long-term follow-up of these patients revealed substantial relapse rates and adverse outcomes: 63 patients (43.8%) experienced relapse, 35 (24.3%) had renal relapse, 13 (9.0%) progressed to ESKD, and 21 (14.6%) died [61, 62]. In the first randomized trial (RAVE) comparing RTX and CYC, relapse at 18 months occurred in 29% of patients in the CYC–AZA group versus 32% in the RTX group, with major relapses reported in 17% and 20%, respectively. Notably, among patients treated with RTX without subsequent maintenance therapy, the relapse rate reached 47% at 18 months [34, 100]. To address this unmet need for safer and more effective treatment strategies, additional randomized controlled trials explored remission maintenance strategies. The French Vasculitis Study Group conducted three randomized maintenance trials, collectively known as the MAINRITSAN studies. In the first trial (MAINRITSAN 1), patients receiving RTX 500 mg every six months had significantly fewer major relapses compared than those receiving AZA (5% vs. 29%) during the first 28 months. Minor relapse rates were 11% and 16%, respectively. Between months 28 and 60, additional major relapses occurred in 23% of RTX-treated patients versus 19% of those in the AZA group, while minor relapses occurred in 12% and 5%, respectively [42, 101]. The second trial (MAINRITSAN 2) compared fixed-schedule RTX with ANCA/B-cell–guided re-treatment. At 28 months, relapse occurred in 17.3% of patients in the tailored-infusion group and 9.9% in the fixed-schedule group, with major relapses in 7.4% and 3.7%, respectively [43]. An international randomized trial (RITAZAREM), which included relapsing patients treated with RTX 1000 mg every four months for two years, showed that RTX was superior to AZA in preventing both any relapse (hazard ratio [HR] 0.41; *p* < 0.001) and major relapses (HR 0.36; *p* = 0.004). Despite this, relapse occurred in 45% of RTX-treated patients (38 patients, with 52 relapses, including 11 major events), indicating a progressive risk of disease recurrence after RTX discontinuation and underscoring the limited durability of its protective effect [31]. Consequently, the third trial (MAINRITSAN 3) investigated extended RTX maintenance (18 additional months) in patients already in sustained remission. Relapse-free survival at 28 months was 96% (95% CI, 91%–100%) in the RTX group versus 74% (95% CI, 63%–88%) in the placebo group, an absolute difference of 22% (95% CI, 9%–36%). Nonetheless, Kaplan–Meier analyses revealed that prolonged therapy for 36 months did not entirely prevent late major relapses (HR 0.69, 95% CI, 0.38 to 1.25) [8]. Furthermore, a recent real-world cohort study reported that approximately 24% of patients relapsed while still receiving RTX maintenance, with 73% of these relapses occurring within the first two years after treatment initiation [91]. These observations underscore the limitations of current RTX-based maintenance strategies and highlight the need for more personalized treatment approaches. Additional insights into maintenance therapy optimization come from the avacopan phase 3 trial. At 52 weeks, relapses were observed in 10.1% of patients treated with avacopan compared with 21.0% in the GC group; although this difference did not reach statistical significance, it suggests a promising trend toward relapse reduction while minimizing GC-related toxicity [26]. Finally, the recent extended maintenance trial (MAINTANCAVAS), with a median follow-up of 4.1 years, compared two re-treatment strategies: RTX upon B-cell repopulation versus RTX upon significant rise in ANCA levels. The relapse rate was 4.1% (95% CI, 1.0–15.6) in the B-cell–guided group compared with 20.5% (95% CI, 11.9–34.1) in the ANCA-driven group [45]. 

Based on evidence from clinical trials and observational studies, accurate risk assessment is essential for the prevention or early detection of relapse in AAV. Accordingly, current guidelines emphasize the need for continuous risk evaluation throughout the disease course, as relapse risk varies substantially among patients [45, 102]. A high-risk relapse phenotype is typically characterized by GPA, PR3-ANCA positivity, ENT and pulmonary manifestations, younger age, infections, a history of previous relapses, persistent or rising ANCA titres during treatment, and seroconversion to ANCA positivity after remission, especially following RTX discontinuation [11, 50, 103–106]. By contrast, a low-risk relapse phenotype is more frequently observed in patients with microscopic polyangiitis, MPO-ANCA–associated disease, severe renal involvement at presentation, older age, and sustained ANCA negativity, particularly in RTX-treated patients with prolonged B-cell depletion. These patients tend to experience fewer relapses and may be candidates for shorter or less intensive maintenance strategies [50, 94, 103, 107]. 

Importantly, patients with MPO-ANCA, older age, and a high burden of comorbidities are frequently underrepresented in randomized controlled trials of AAV, which have largely enrolled younger and PR3-ANCA positive patients. In Western cohorts, these characteristics often overlap and correspond to MPA, a phenotype that is typically monophasic but affects older patients who are inherently more frail and infection-prone [94, 108]. Moreover, the inclusion of different AAV subtype in the same study, often necessary to achieve adequate sample sizes in rare diseases, may limit the translation of results into routine clinical practice, given differences in clinical presentation, disease course, and relapse patterns between GPA and MPA. In addition, ANCA-negative patients, who account for approximately 10–20% of patients with GPA and are more frequently observed in localized forms of disease, remain underrepresented [109]. 

Taken together, these findings highlight the continuing need for innovative therapeutic strategies and predictive tools to achieve durable remission and reduce relapse risk [94]. 

### Refractory Manifestations

Certain disease manifestations remain resistant to standard treatment, particularly granulomatous inflammation, which is a hallmark of GPA [110–112]. As shown in Table 3, granulomatous manifestations primarily affect the respiratory tract, including the upper airways (nose, paranasal sinuses, and middle ear), lungs, orbit, and, less commonly, the kidneys and central nervous system, where hypertrophic pachymeningitis can occur [76, 111, 113–118]. The relative paucity of vasculitic features, together with the distinct inflammatory milieu present in granulomatous lesions and the presence of fibrinoid necrosis, contributes to the suboptimal response to conventional immunosuppressive therapies [117, 119]. A retrospective study involving 59 patients with refractory GPA revealed that RTX achieved complete remission in only 9.3% of cases. Notably, while a favourable response was observed in 89.2% of patients with renal involvement, it was observed in only 44.4% of those with orbital granulomas. Relapse rates were high (40%) despite maintenance treatment [110]. A multicentre retrospective study involving 80 patients, conducted by the French Vasculitis Study Group, further confirmed the limited efficacy of standard treatment in patients with granulomatous manifestations [120]. Furthermore, although subglottic stenosis is an uncommon manifestation of AAV, it poses significant therapeutic challenges and may be life-threatening [118, 121]. The efficacy of local interventions, including surgical and endoscopic procedures, as well as systemic immunosuppressive treatments in achieving sustained disease control is limited. Patients often experience refractory symptoms and frequent relapses [118, 121]. Finally, among granulomatous manifestations, pseudotumor-like lesions represent a rare but particularly challenging subtype. These masses may mimic malignancies or abscesses. Despite the administration of immunosuppressive therapy, a significant proportion of these lesions have proven resistant to treatment [117]. In selected cases, mass excision was deemed necessary, although relapses occurred after surgery. These findings emphasise the limitations of conventional immunosuppressive regimens, reinforcing the concept that granulomatous inflammation in GPA may respond differently from vasculitic components and may require distinct management strategies [115, 117].

**Table 3:** Management strategies for refractory manifestations

Manifestation	Systemic disease control	Local/procedural measures	Monitoring & key points
Subglottic stenosis/airway obstruction	RTX re-induction; consider short CYC if rapidly progressive	Endoscopic dilation/laser; intralesional steroids	Pair local with systemic therapy; schedule airway follow-up (symptoms, endoscopy, imaging).
Sinonasal granulomatous inflammation	RTX re-induction or MTX/MMF for milder courses	Endoscopic debridement, saline irrigations; culture-directed antibiotics if superinfection	Reassess with nasal endoscopy; avoid overtreating stable damage/scarring without inflammatory activity.
Orbital mass/pseudotumor	RTX re-induction; short CYC if aggressive compressive disease	Image-guided biopsy in case of atypical features	Urgent ophthalmology input if optic compromise; monitor with MRI/CT.
Pulmonary nodules/cavities	RTX re-induction; consider short CYC for threatening disease	Manage cavitary complications; bronchoscopic sampling if infection suspected	Rule out infection before treatment escalation; serial CT in complex cavities.

*CT* computed tomography, *CYC* cyclophosphamide, *MMF* mycophenolate mofetil, *MRI* magnetic resonance imaging, *MTX* methotrexate, *RTX* rituximab

Finally, drug-induced vasculitis is a complex and difficult-to-treat subset that closely mimics idiopathic AAV in both clinical and serological features. Several agents have been implicated in its pathogenesis, including hydralazine, propylthiouracil, minocycline, and particularly cocaine, especially when adulterated with levamisole [122, 123]. 

## Emerging Biomarkers Beyond ANCA and B-Cell Repopulation to Support a Personalized Therapeutic Approach

Beyond ANCA specificity and B-cell–related parameters, several emerging biomarkers are under investigation to refine disease activity assessment and support a more personalized therapeutic approach in AAV [124, 125]. Among these, markers of complement activation have gained particular relevance. Several observational studies have shown that low circulating C3 levels at diagnosis are associated with higher disease severity, worse renal function, and poorer renal and overall survival [126–128]. Recently, low circulating C4 levels have also been proposed as a prognostic biomarker in patients with MPA [129]. Beyond systemic measurements, intra-glomerular (factor B) and urinary (Bb, C3a, C5a, sC5b-9) markers of alternative pathway activation may better reflect ongoing inflammatory renal injury, although evidence remains inconsistent across studies [130–132]. In parallel, urinary soluble CD163 (usCD163) represents one of the most robust emerging marker of active renal involvement in AAV [133–137]. CD163 is a haemoglobin–haptoglobin scavenger receptor expressed on activated monocytes and M2 macrophages and is released in soluble form during inflammatory responses [137]. Across multiple studies, usCD163 has demonstrated high sensitivity and specificity for the detection of active renal vasculitis, distinguishing inflammatory activity from chronic damage, and predicting renal disease relapse [133–137]. 

Another urinary renal biomarker is monocyte chemoattractant protein-1 (MCP-1, also known as CCL2), which has shown potential as a marker of active renal involvement in AAV. Several studies have reported increased urinary MCP-1 levels during renal relapse, associations with disease severity and worse renal outcomes, and a decline in response to effective treatment [138–141]. Furthermore, in a longitudinal multicentre cohort, the combination of usCD163 and usMCP-1 improved the identification of renal vasculitis relapse compared with either biomarker alone, supporting the added diagnostic value of integrating complementary macrophage-derived urinary markers in AAV [142]. 

A post-hoc analysis of samples collected during one of the largest trials (RAVE) showed that elevated interleukin-6 (IL-6) levels correlated positively with ANCA titres in patients with PR3-ANCA, but not in those with MPO-ANCA [143]. 

Finally, several additional candidates are under investigation to enable a more precise and individualized assessment of AAV, encompassing disease activity, organ involvement, and prognosis, and thereby providing a biological framework to support the development and contextualization of emerging targeted therapies [124]. 

## Emerging Pharmacological Therapies with Clinical Evidence in AAV

**Fig. 1:**
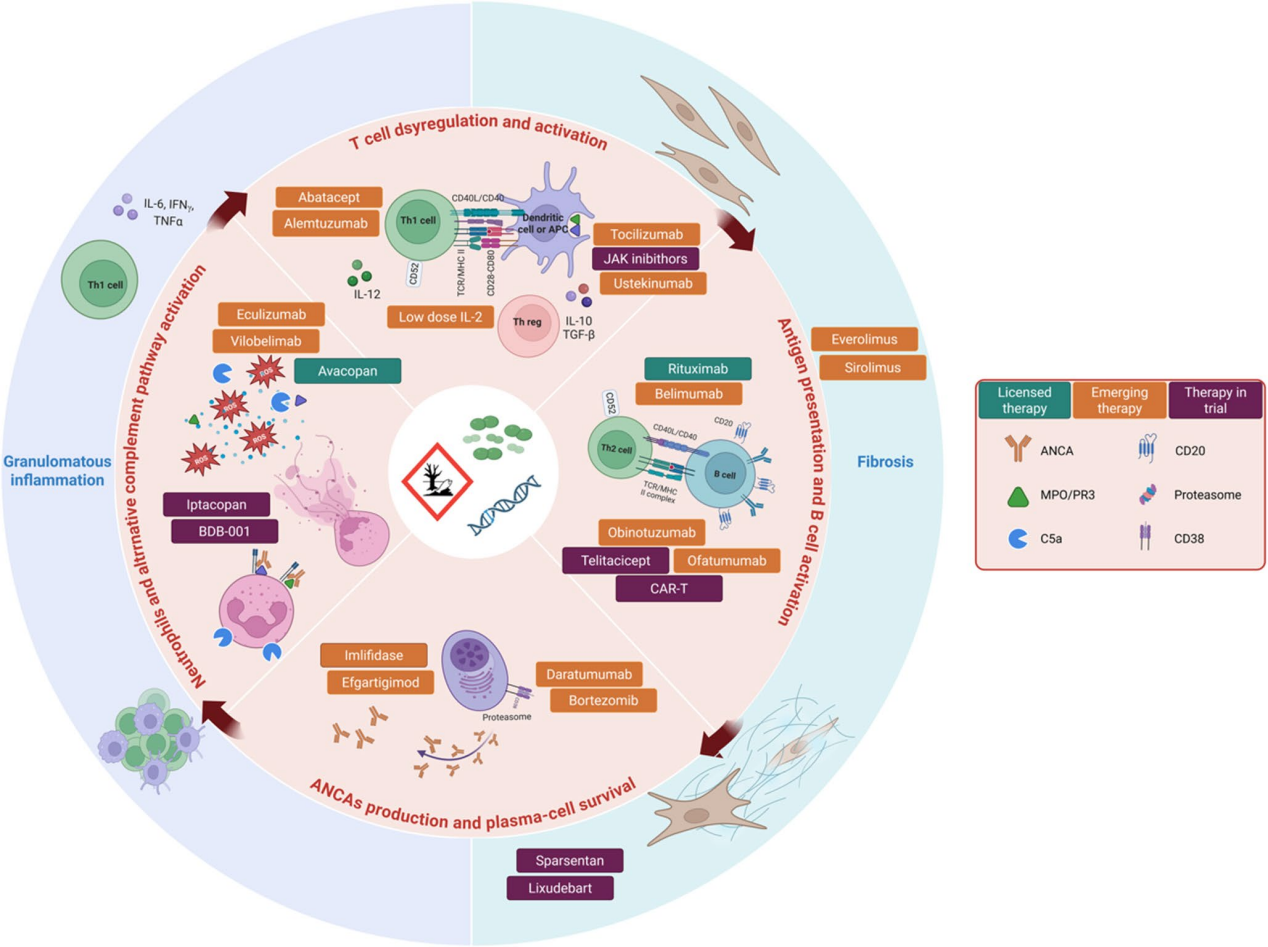
Therapeutic targets and emerging treatments in ANCA-associated vasculitis (AAV)

Despite the progress and increasing number of studies in recent years, new treatment modalities are needed, especially in patients who are refractory, intolerant, or frequently relapse despite standard immunosuppressive treatment, in particular to avoid or at least reduce the still unacceptably high short- and long-term treatment-related toxicity. Targeted therapies are shown in Fig. 1.

Schematic representation of the major immunopathogenic pathways involved in AAV, together with the therapeutic agents that are currently in use or under investigation. The diagram highlights interventions targeting T-cell dysregulation and activation; antigen presentation and B-cell activation; ANCA production and plasma-cell survival; neutrophil activation and the alternative complement pathway; and granulomatous inflammation. Licensed therapies, emerging molecules, and agents currently in clinical trials are shown according to their specific immunological targets.

### Targeting B and Plasma Cells

B cells play a central role in AAV pathogenesis. Beyond serving as precursors to ANCA-secreting plasma cells, they also function as professional antigen-presenting cells, efficiently presenting antigens to autoreactive T cells and delivering co-stimulatory signals essential for T cell activation. Their pathogenic involvement is supported by the presence of B cells in granulomatous lesions [144], by the correlation between B-cell numbers and disease activity [35], and by the association between B-cell repopulation after depletion therapy and an increased risk of relapse [11]. The introduction of RTX has transformed AAV management, offering an effective alternative to CYC in both remission induction and maintenance phases [12]. Other CD20-targeting B-cell depleting antibodies are currently used in the treatment of haematological and other autoimmune diseases. Furthermore, these agents have shown efficacy in cases of RTX-resistant disease and in patients with allergic reactions to RTX [145–149]. Some of these newer monoclonal antibodies are fully human, a property that may help circumvent immunogenic anti-drug reactions. Therapeutic positioning and sequencing of emerging B-cell–directed therapies in AAV remain largely guided by expert opinion rather than comparative evidence. RTX is still the cornerstone of B-cell–targeted treatment, while alternative anti-CD20 agents are generally reserved for specific clinical scenarios. Inhibition of the BAFF/APRIL pathway represents a biologically plausible adjunctive strategy, particularly in light of the post-RTX BAFF surge; yet, evidence supporting sequential or combination approaches is currently derived from other immune-mediated diseases [150], and comparable data in AAV are lacking. Consequently, therapeutic decisions should be individualized based on disease phenotype, relapse pattern, prior treatment response, and treatment-related toxicity. Prospective sequencing trials remain an important unmet need.

From a safety perspective, serious infections remain the main concern associated with B-cell–directed therapies in AAV, as they represent the leading cause of mortality, particularly during the first year of RTX treatment [80]. Hypogammaglobulinemia occurs more frequently in patients with low baseline IgG levels, high GC exposure, and older age, and IgG levels < 6 g/L are strongly associated with an increased risk of serious infections [91, 151]. These observations support routine immunoglobulin monitoring and infection-prevention strategies. At present, safety data for newer B-cell–targeted agents in AAV remain limited, and their long-term infectious risk requires further study.

#### Ofatumumab

Ofatumumab is a fully human type I anti-CD20 monoclonal antibody targeting a distinct extracellular epitope of CD20. Compared with RTX, it demonstrates slower dissociation kinetics (lower “off-rate”) and enhanced complement activation potential, resulting in stronger cytotoxic effects, particularly in cases with low CD20 expression levels [145]. A case series comprising eight AAV patients, including EGPA, MPA, and GPA described the efficacy and safety profile of ofatumumab [152]. In this study, a combination of ofatumumab, GCs, and low-dose CYC produced serological and clinical responses similar to those observed in other cohorts. Interestingly, among the eight ofatumumab-treated patients, one patient with GPA had previously experienced an anaphylactic infusion reaction to RTX, while another EGPA patient had previously received RTX [152]. 

#### Obinutuzumab

Obinutuzumab is a humanized type II anti-CD20 monoclonal antibody. Compared with RTX, obinutuzumab induces more profound and longer-lasting B-cell depletion, exhibits increased binding to FcγRIIIA and enhances natural killer (NK)-mediated antibody-dependent cell-mediated cytotoxicity (ADCC), leading to direct cell death induction [145]. A phase 3 randomized controlled trial (REGENCY) recently demonstrated that the combination of obinutuzumab and standard therapy was superior to standard therapy alone in achieving complete renal response in adults with active lupus nephritis [149]. A small, single-centre case series (*n* = 3) has explored obinutuzumab treatment in refractory AAV patients [153]. All three patients had a history of relapsing disease and had previously received RTX, which resulted in anaphylactic infusion reactions during the second cycle. Obinutuzumab was successfully administered to these patients for both remission induction and maintenance. Currently, three prospective multicentre studies are investigating obinutuzumab as an induction treatment for AAV patients: a French open-label study (OBI-WAN) in relapsing patients, a British study (ObiVAS) [154] and a US controlled trial (NCT05376319) comparing obinutuzumab with RTX.

#### Bortezomib

Bortezomib, a proteasome inhibitor approved for multiple myeloma, selectively depletes plasma cells with high immunoglobulin synthesis [155]. Its mechanism of action involves blocking activation of the anti-apoptotic nuclear factor kappa B (NF-κB) pathway and disrupting protein homeostasis within the endoplasmic reticulum. This leads to the accumulation of misfolded proteins, which ultimately induce apoptosis [156]. Due to their exceptionally high antibody production rate, plasma cells are particularly vulnerable to proteasome inhibition. Preclinical studies have demonstrated the potential of bortezomib in autoimmune diseases, including systemic lupus erythematosus (SLE), in which it prevented the development of glomerulonephritis in mouse models [157]. Similarly, in a murine model of ANCA-associated nephritis, bortezomib significantly reduced MPO-specific plasma cells, leading to lower MPO-ANCA levels and attenuation of glomerulonephritis [158]. Although clinical data remain scarce, a case report described a patient with refractory PR3-AAV who achieved sustained remission of kidney disease after a single cycle of bortezomib, allowing GC discontinuation [159]. Despite its potential, broader use in autoimmune diseases may be constrained by its safety profile, particularly the high incidence (> 30%) of painful peripheral neuropathy, although lower doses than those used in oncology might mitigate this risk [160]. 

#### Belimumab

Targeting B-cell Activating Factor (BAFF), a key regulator of B-cell survival and activation, may represent a promising therapeutic strategy for AAV. Elevated BAFF levels have been observed in patients with GPA and MPA [161, 162], and ANCA-activated neutrophils may contribute to its release [163]. Following B-cell depletion with RTX, BAFF concentrations rise further [164], potentially driving the positive selection of autoreactive B cells during immune reconstitution and increasing the risk of relapse [165, 166]. Belimumab, a fully human monoclonal antibody that selectively binds soluble BAFF, prevents its interaction with B-cell receptors, thereby reducing B-cell survival and promoting apoptosis. Originally approved for SLE, it has also been investigated in AAV. A phase 3 trial (BREVAS - Belimumab in Remission of Vasculitis trial) evaluated belimumab as a maintenance therapy alongside AZA and GCs in AAV patients following induction with RTX or CYC [167]. The trial was prematurely terminated due to recruitment challenges, limiting its statistical power. Although no significant difference in overall relapse rates was observed, patients with RTX-induced remission who were subsequently treated with belimumab exhibited no relapses. A randomised, controlled trial (NCT03967925) was designed to investigate the combination of belimumab and RTX in PR3-positive patients and was expected to conclude in November 2023, but no results have been reported to date.

#### Daratumumab

The limited efficacy of standard immunosuppressive therapies is partly due to the persistence of long-lived plasma cells that remain unaffected by conventional treatments. These cells evade targeting due to their lack of CD20 expression and their ability to survive within niches in the bone marrow [168, 169]. By continuing to produce ANCA, these plasma cells perpetuate the autoimmune process. Targeting CD38, a molecule highly expressed on long-lived plasma cells, represents a potentially effective therapeutic strategy. Daratumumab, an anti-CD38 monoclonal antibody approved for multiple myeloma, has demonstrated successful application in SLE, haemolytic anaemia, and immune thrombocytopenia. Recent literature has documented three cases of severe refractory AAV successfully treated with daratumumab as add-on therapy [170, 171]. 

#### CAR T Cells

An additional emerging strategy to achieve broader B-cell depletion is targeting CD19, which is expressed throughout B-cell maturation, from early precursors to plasmablasts, making it a promising candidate for more comprehensive immunomodulation. CD19-directed chimeric antigen receptor (CAR) T-cell therapy, designed to eliminate pathogenic B cells, offers notable advantages over conventional agents like RTX, including the ability to migrate to and persist within lymphoid and target organs, thereby ensuring prolonged immune control [172]. 

In a murine MPO-AAV model, CD19 CAR-T cells achieved sustained depletion of B cells and plasmablasts in peripheral blood, spleen, bone marrow, and kidneys [173]. Although MPO-ANCA levels were not completely abolished, they were significantly reduced, and treated mice were protected from necrotizing crescentic glomerulonephritis, with no histological evidence of this complication. These preclinical findings support the potential of CD19 CAR-T cells to induce and maintain drug-free remission in AAV. Early-phase clinical trials investigating CAR-T cell therapy in autoimmune diseases (including AAV) are currently underway, with a phase 1/2 trial (NCT06590545 - IDEAL) specifically evaluating its application in AAV.

### Targeting T Cells

The relative paucity of therapies directly targeting T cells in AAV may indicate that interventions aimed at complement blockade and autoantibody depletion exert more rapid and substantial effects by suppressing neutrophil activation at the endothelial surface, resulting in stronger outcomes in preclinical and early-phase clinical studies. Nonetheless, dysregulated T cell responses remain central to AAV pathogenesis. It is widely recognized that T cells drive B cell activation, IgG class switching, and neutrophil-mediated tissue damage. Among CD4 + T cell subsets, Th17 cells dominate during active disease, promoting inflammation through IL-17 and IL-23 signalling [174], while regulatory T cells (Tregs) are impaired, potentially causing a loss of immune tolerance and favoring autoimmune responses [175]. Furthermore, an expanded population of circulating effector memory CD4 + T cells may contribute to disease chronicity [176]. CD8 + T cells exacerbate the disease by enhancing neutrophil activation through MHC class II induction [177], and their depletion in experimental models reduces glomerular injury [178]. Notably, T cell activation persists even during remission, indicating that therapies targeting T cells might be effective in preventing relapses and achieving better long-term disease control [179]. 

#### Alemtuzumab

Alemtuzumab, a humanized monoclonal antibody targeting CD52, induces profound depletion of circulating lymphocytes and monocytes, with particularly long-lasting effects on T cells. CD4 + T cell recovery is slow, often remaining incomplete for months to years [180]. However, Tregs tend to increase, contributing to prolonged immunomodulatory effects [181]. Originally approved for lymphoma, alemtuzumab has also been used off-label in hematopoietic stem cell transplantation and is licensed for relapsing-remitting multiple sclerosis. Given its potent immunosuppressive effects, it has been explored as a treatment for refractory AAV. In a retrospective study and a subsequent dose-ranging trial (ALEVIATE), alemtuzumab induced remission in the majority of AAV refractory patients. Despite this, relapses were frequent and often required re-treatment [182, 183]. Additionally, severe adverse events including infections, malignancies, and thyroid disorders were common, raising concerns about its long-term safety. Although alemtuzumab may provide a therapeutic option for patients with difficult-to-treat AAV, its high relapse rate and significant safety concerns limit its broader clinical application.

#### Abatacept

Abatacept, a fusion protein combining the Fc region of IgG1 with the extracellular domain of cytotoxic T lymphocyte antigen 4 (CTLA-4), blocks T cell activation and disrupts B-T cell crosstalk, attenuating pathogenic immune responses. By inhibiting T cell co-stimulation, it prevents CD28-CD80/CD86 interactions on antigen-presenting cells, including B cells [184]. Originally approved for rheumatoid arthritis (RA), abatacept was tested in patients with non-severe relapsing GPA.

In a phase 1/2 trial (NCT00468208) involving 20 patients with relapsing, non-severe GPA, abatacept was administered intravenously for six months in combination with methotrexate, mycophenolate mofetil, or azathioprine [185]. The treatment was generally well tolerated and showed a favourable safety profile. Clinical improvement was observed in 90% of patients, as indicated by a reduction in Birmingham Vasculitis Activity Score (BVAS)/Wegener (WG) scores, and 80% achieved disease remission. Notably, abatacept also demonstrated steroid-sparing properties. However, six patients discontinued the study due to disease worsening, including three who had initially achieved remission but later relapsed. A separate placebo-controlled phase 3 trial (NCT02108860 - ABROGATE) evaluated subcutaneous abatacept in 65 patients with relapsing, non-severe GPA [186]. Participants received abatacept or placebo alongside a GC taper and stable background immunosuppressive therapy. Results showed that the difference in treatment failure rates between abatacept and placebo was not statistically significant. Abatacept also failed to show superiority on key secondary endpoints, including time to remission and GC-free disease control. Nevertheless, abatacept was generally well tolerated, with a safety profile comparable to that of placebo.

### Complement Inhibition

Although AAVs are traditionally considered ‘pauci-immune’ diseases, histopathological studies have identified complement deposition in inflamed tissues, including C3 and terminal complement components in glomeruli [187]. Complement activation is further supported by altered plasma and urinary complement levels, which correlate with disease severity [132]. Experimental models highlight the role of the alternative pathway and C5a in driving neutrophil recruitment and endothelial damage, making complement inhibition a promising therapeutic strategy [188]. As mentioned above, targeting C5a signalling has shown clinical benefits, leading to the development of avacopan [26, 189]. 

#### Eculizumab

Several case reports have investigated eculizumab as a combination treatment for AAV, yielding preliminary results worthy of further exploration [190–193]. Eculizumab is a humanised anti-C5 monoclonal antibody approved for the treatment of paroxysmal nocturnal haemoglobinuria and atypical haemolytic uremic syndrome. It has also shown efficacy in treating antiphospholipid syndrome [194]. 

#### Vilobelimab and Iptacopan

Vilobelimab (IFX-1) is a monoclonal antibody that selectively targets C5a, effectively inhibiting neutrophil activation, chemotaxis, and complement-mediated inflammation while preserving C5 cleavage and membrane attack complex formation, thus differentiating it from avacopan, which blocks C5aR [195, 196]. It has been investigated across multiple conditions, including hidradenitis suppurativa, pyoderma gangrenosum, COVID-19, and sepsis [195–198]. In AAVs, a phase 2 trial (NCT03712345) confirmed its favourable safety profile, though efficacy data were inconclusive due to early termination during the COVID-19 pandemic [199]. A subsequent trial (IXCHANGE) further explored its role in AAV treatment, revealing promising GC-sparing potential. Patients receiving vilobelimab had a markedly lower GC toxicity index and fewer treatment-emergent adverse events compared with standard GC regimens. A randomized controlled study (NCT06388941) is currently underway to investigate the effects of ipatacopan , a small molecule designed to bind to factor B of the alternative complement pathway and regulate C3.

### Other Therapeutic Strategies

#### JAK- Inhibitors

The Janus kinase (JAK)/signal transducer and activator of transcription (STAT) pathway is a key mediator of inflammatory and immune responses. Several cytokines, including IL-6, IL-10, IL-12, IL-23, and type I interferons, contribute to AAV pathogenesis by activating this signalling cascade [200, 201]. Upon cytokine binding to their receptors, JAKs phosphorylate STATs, which dimerize and translocate to the nucleus, triggering gene transcription. Given the central role of this pathway in autoimmunity, JAK inhibitors have emerged as targeted therapeutic options. Tofacitinib, a JAK1/3 inhibitor, has shown efficacy in autoimmune diseases such as RA by modulating T cell responses [202]. Its potential in AAV has been explored in small studies, in which most patients with GPA or MPA achieved remission or clinical improvement [203, 204]. Nevertheless, some patients discontinued treatment due to insufficient efficacy or adverse events, including a case of pulmonary embolism. Larger studies are needed to clarify its efficacy and safety across different disease manifestations.

#### Everolimus/Sirolimus

One of the most challenging manifestations of GPA is granulomatous disease, which likely represents a pathogenetically distinct subgroup that proves particularly difficult to treat [111, 116, 205]. Extravascular granulomatous inflammation presents a variable histopathological picture of inflammation, fibrinoid necrosis, and excessive fibrosis [111]. In preclinical studies, sirolimus, a mammalian target of rapamycin (mTOR) inhibitor, has demonstrated the ability to reduce fibroblast proliferation and collagen expression [206, 207]. In a retrospective review of a cohort of patients with subglottic stenosis (SGS), sirolimus was administered to explore its immunosuppressive and antifibrotic effects [208]. Six patients were treated, five of whom had GPA; four of six tolerated sirolimus without significant adverse events and achieved a steroid-sparing effect. Consistently, a case report described sustained improvement of tracheal stenosis secondary to AAV after sirolimus initiation, supporting its potential role in airway inflammatory disease [209]. Recently, a phase 1 non-randomised clinical trial demonstrated the safety of everolimus, another mTOR inhibitor, in eight subjects with idiopathic SGS, while also maintaining peak expiratory flow [210]. 

#### Lixudebart

Lixudebart (ALE.F02/ALE.01) is a first-in-class monoclonal antibody that binds an exposed non-junctional claudin-1 epitope, aiming to reverse organ fibrosis across the liver, lung and kidney [211]. Non-junctional CLDN1 upregulation in crescents may contribute to rapidly progressive glomerulonephritis [212]. In renal AAV, the ongoing, randomized, double-blind, placebo-controlled trial (NCT06047171 - RENAL-F02) has dosed 26 patients with ANCA-rapidly progressive glomerulonephritis for up to 24 weeks on top of standard of care. Interim topline data indicate dose-dependent target engagement, a favourable safety profile, and signals of renal benefit including improved eGFR recovery and reduced proteinuria, accompanied by reductions in usCD163 [213]. While these findings support the therapeutic plausibility of CLDN1-directed anti-fibrotic therapy as an adjunct to immunosuppression in AAV, they remain interim and sponsor-reported; peer-reviewed analyses and longer-term outcomes (dialysis-free survival and ESKD) are awaited.

#### Tocilizumab

Clinical evidence supports the hypothesis of a central role of IL-6 in AAV pathogenesis [143, 214, 215]. IL-6, a major differentiation factor for the B cell lineage, appears elevated in serum and biopsies of active AAV patients. The high inflammatory burden of AAV and the available clinical evidence have led to case reports documenting the efficacy of tocilizumab, a monoclonal antibody targeting the IL-6 receptor [205, 215–217]. A small, single-arm Japanese study suggested that tocilizumab monotherapy may offer an alternative treatment strategy for some MPA patients [218]. Of six patients treated with intravenous tocilizumab monotherapy without GC, two (33.3%) achieved complete remission and four (66.7%) achieved partial remission at month 6 (with one patient voluntarily discontinuing treatment at month 3). A larger clinical trial (JMAIIA00325) has been announced to evaluate the efficacy, safety, and pharmacokinetics of intravenous tocilizumab plus GC in patients with active MPA and GPA compared with CYC plus GC [219]. 

#### Ustekinumab

A recent case series demonstrated the efficacy of ustekinumab, a monoclonal antibody targeting IL-12 and IL-23, combined with GC and CYC, in patients with relapsing AAV [220]. The choice of ustekinumab was based on spatial and single-cell transcriptome analyses characterising inflammatory niches in kidney samples from enrolled patients. Four patients with relapsing AAV received ustekinumab in combination with low-dose CYC and steroids. All patients tolerated the treatment well, and clinical responses were observed in all cases.

#### Low Dose IL-2 Therapy

IL-2 is a T cell growth factor with pleiotropic functions. The dose of IL-2 has been hypothesised to be a driver of the imbalance between autoimmunity and immune tolerance. The administration of low-dose IL-2 has emerged as a promising approach for inducing regulatory T cells (Treg), offering potential therapeutic benefits in the treatment of various autoimmune diseases [221–223]. Two distinct cytological studies have demonstrated that low-dose IL-2 therapy leads to an increase in the peripheral blood Treg cell population in patients with active AAV [223, 224]. A phase 1/2 study involving 46 patients diagnosed with various autoimmune diseases, including GPA, confirmed specific Treg expansion. Furthermore, low-dose IL-2 was well tolerated, regardless of the disease and concomitant treatments [221]. Currently, there are no ongoing phase 2 or 3 studies investigating this therapy in AAV patients.

#### Anti-Immunoglobulin Agents

Efgartigimod, a humanised IgG1 Fc fragment, binds to the neonatal Fc receptor (FcRn) and inhibits its interaction with IgG. This mechanism reduces IgG recycling and promotes degradation of pathogenic autoantibodies [225]. A case report describes the successful treatment of severe refractory cutaneous involvement in an AAV patient using efgartigimod as add-on therapy [226]. Efgartigimod has been approved for the treatment of myasthenia gravis. Two years ago, the pharmaceutical company announced a clinical trial investigating its use in AAV. However, the continuation of the trial has recently been questioned [225]. 

Imlifidase is a cysteine protease derived from Streptococcus pyogenes that works by cleaving IgG into F(ab′)2 and Fc fragments, thereby inhibiting its role in complement-dependent cytotoxicity (CDC) and ADCC [227, 228]. In a single-arm phase 2 study (EudraCT 2016–004082-39), patients with severe anti-GBM disease treated with imlifidase showed a reduction in circulating anti-GBM antibodies [229]. Imlifidase was also used in two cases of severe refractory AAV, one associated with anti-GBM antibodies [228, 230]. A single-centre 24-week phase 2 trial (2021–004706-22 - ImlifidARDSe) evaluating the efficacy of imlifidase plus standard of care for the treatment of AAV with severe diffuse alveolar haemorrhage is still ongoing.

### Ongoing Trials and New Therapeutics on the Horizon

The pipeline of novel therapeutics is encouraging; however, uncertainties remain regarding optimal sequencing, combination with current standards, and applicability beyond trial populations. Table 4 summarizes the agents currently under investigation in ongoing AAV trials for which results have not yet been reported.

**Table 4:** Agents currently under investigation in AAV in trials

Trial registration number	Drug	Mechanism of action	Rationale	Administration route	Design	Population (n)	Primary outcome	Status	Trial duration	Estimated study completion date	Intended use of the experimental drug
NCT05197842	BDB-001	anti-C5aR1 mAb activating TLR 7/8	C5a inhibition to reduce neutrophil activation and vascular damage	IV	Multicentre, randomized, open-label, parallel-controlled, phase 1/2	100	Complete or partial remission by BVAS (time frame: 12 weeks)	Recruiting	24 weeks	2025-03	Induction of remission (substitution of GCs with BDB-001 injection)
NCT05962840	Telitacicept	BAFF/APRIL dual-target-inhibitor	- Efficacy in other rheumatic diseases - Efficacy of similar drugs in AAV	SC Injection	Single-centre, prospective, open-label, randomized, controlled, phase 4	40	Time to relapse (time frame: 24 months)	Recruiting	24 months	2026-12-31	Induction of remission (remission rate of telitacicept + RTX vs. telitacicept alone)
NCT05965284	Telitacicept	BAFF/APRIL dual-target-inhibitor	- Efficacy in other rheumatic diseases; - Efficacy of similar drugs in AAV	SC Injection	Single-centre, prospective, open-label, randomized, controlled, phase 4	40	Time to relapse (time frame: 12 months)	Recruiting	12 months	2026-12-31	Remission-maintenance (remission rate of telitacicept + AZA vs. AZA alone)
NCT06656962	Telitacicept	BAFF/APRIL dual-target-inhibitor	- Efficacy in other rheumatic diseases - Efficacy of similar drugs in AAV	SC Injection	Single-centre, prospective, single-arm, open-label, phase 1/2	15	Complete remission of AAGN by BVAS (time frame: 24 weeks)	Active, not recruiting	48 weeks	2026-10-30	Induction of remission (efficacy of telitacicept + GC and CYC in AAGN)
NCT06590545	KYV101 (CAR T cell therapy)	Fully human anti-CD19	- Efficacy in other rheumatic diseases - Efficacy of B cell depletion	IV	Two-stage interventional, prospective, open-label, phase 1/2	8	- Phase 1, safety: n. of CRS, ICANS, AE and SAE (time frame: 4 weeks) - Phase 2, efficacy: ANCA seroconversion (time frame: 24 weeks); safety: n. of AE and SAE (time frame: 52 weeks)	Not yet recruiting	52 weeks	2027-07	Induction of remission in active, treatment refractory, ANCA-IgG-positive vasculitis
NCT07160608	Tarperprumig (ALXN182)	Suppression of the alternative complement pathway through inhibition of properdin	Efficacy of complement inhibition in AAV	SC Injection	Multicentre, randomized, double-blind, placebo-controlled, parallel-group, phase 2	75	Safety: n. of participants with treatment-emergent adverse events	Recruiting	70 weeks	2028-02-14	Induction of remission
NCT06388941	LNP023 (Iptacopan)	Inhibition of alternative complement pathway binding Factor B	Efficacy of complement inhibition in AAV	Oral	Multicentre, randomized, controlled, phase 2	78	Remission without major relapse (time frame: 48 weeks)	Recruiting	48 weeks	2027-10-05	Induction of remission (efficacy of Iptacopan + RTX)
NCT04944524	Tofacitinib	JAK1 and JAK2 inhibitor	- Efficacy in other rheumatic diseases - Efficacy in small case series in AAV	Oral	Single-centre, randomized, phase 4	66	Relapse rate (time frame: 12 months)	Unknown status	12 months	2024-07-01	Remission maintenance in GPA (Tofacitinib vs. MTX)
2021–004706-22	Imlifidase	IgG cleaving cysteine protease	- Efficacy of similar drugs in AAV - Efficacy in small case series in AAV	IV	Prospective, single-arm, open-label, phase 2	10	ANCA seroconversion (titre below reference range) within 24 h of administration	Unknown status	24 weeks	-	Induction of remission (efficacy of imlifidase + SoC in severe AAV with pulmonary haemorrhage)
NCT06047171	Lixudebart (ALE.F02)	anti-CLDN11 mAb	Efficacy in other diseases: protect vascular integrity, halt fibrosis	IV	Multicentre, randomized, double-blind, placebo-controlled, phase 2	80	Safety and tolerability (time frame: 52 weeks)	Active, not recruiting	52 weeks	2025-09-25	Protect and preserve kidney function in RP AAGN (Lixudebart + SoC)
NCT05630612	Sparsentan	Endothelin-A receptor and angiotensin II-1 receptor blocker	Improvement of vessel stiffness and fibrinolytic capacity by endothelin receptors blockade	Oral	Single-centre, randomized, double-blind, active control, parallel-group study, phase 2	32	Change in ACh-mediated forearm blood flow vasodilatation (from baseline to week 6)	Active, not recruiting	6 weeks	2027-09-01	Improvement of endothelial function in patients in remission (sparsentan vs. irbesartan)

*AAGN* ANCA-Associated Glomerulonephritis, *AAV* ANCA-Associated Vasculitis, *ACh* Acetylcholine, *AE* Adverse Events, *AZA* Azathioprine, *CLDN1 *Claudin 1, *CRS* Cytokine Release Syndrome, *CYC* Cyclophosphamide, *GCs* Glucocorticoids, *GPA* Granulomatosis with Polyangiitis, *ICANS* Immune Cell-associated Neurotoxicity Syndrome, *Ig* Immunoglobulin, *JAK* Janus Kinase, *mAb* monoclonal Antibody, *RP* rapidly progressive, *RTX* Rituximab, *SoC* Standard of Care, *SAE* serious adverse events, *TLR* Toll Like Receptor

## Conclusions

AAV is a life-threatening, multisystem disease in which early diagnosis and rapid, organ-protective therapy are critical. For remission induction, RTX or CYC remain the backbone, combined with GC minimization (reduced-dose schedules; avacopan where steroid toxicity is a priority). In severe kidney disease (i.e. rapidly progressive glomerulonephritis), adjunctive PLEX may be considered selectively after an individualized risk–benefit discussion and combining RTX with low-dose CYC is a reasonable option in highly active presentations. For remission maintenance, fixed-interval RTX is preferred over conventional oral agents, particularly in PR3-ANCA disease, yet relapses still occur after cessation. Thus, management should incorporate structured monitoring and a clear plan for the early detection and timely treatment of relapses.

Emerging agents are likely to complement, rather than replace, current induction and maintenance strategies. Looking ahead, the integration of biomarker-driven therapy, precision medicine approaches, and patient-centred outcomes will further refine AAV management, moving the field toward strategies with minimal treatment burden and optimized long-term outcomes.

## Data Availability

No datasets were generated or analysed during the current study.
